# Clinic Atlanta Medical College

**Published:** 1876-06

**Authors:** A. W. Calhoun

**Affiliations:** Professor of Diseases of the Eye and Ear


### Clinic Atlanta Medical College.

A. W. CALHOUN, M.D., Professor of Diseases of the Eye and Ear.

(*Continued from page 24 of April Journal.*)

CASE XLIII.—*Double Cataract existing for twenty-two years in a young man. Apparently soft, but found to be perfectly hard. Restoration of vision in one eye; a negative result in the other.*—T. J. W., æt. thirty, Monticello, Ga., had good sight up to his seventh year, at which time it began to fail, and was for all practical purposes completely lost at the expiration of his eighth year, only a good perception of light remaining. He has a clear recollection of seeing in his childhood, the last time being on the occasion of riding to a blacksmith shop, when seven years old, and seeing the smiths shoe his horse. Only this one incident is distinct in his memory, all others being undefined, and his recollection of them being clouded and uncertain. His sense of touch has become unusually acute, and his vision, so to speak, has been transferred to the ends of his fingers. He is delicate in appearance, yet has had perfect health throughout his entire life. There is nothing hereditary in his condition, so far as can be gained from his history.

After dilating the pupils, the cataract in each eye presented all the evidences of being soft, as is usually found in the young. The perception of light was good, and he was able to distinguish the prominent colors one from the other—a circumstance, however, of but little practical value, more particularly in cases of congenital cataract; for I have not unfrequently observed that even where tolerably minute shades of color were at once distinguished by children thus affected, the complete removal of the opaque lens (cataract), left them in about the same condition as prior to any surgical interference. This was due, no doubt, to the long disuse of the optic nerve and retina.

Believing the cataract to be soft, the needle operation (laceration) was made through the cornea, in the right eye, but the needle penetrated only the anterior capsule of the lens, and broke up a small portion of the cortical substance, the main portion of the lens resisting the cutting or lacerating process. On the day following the operation, violent inflammation began around the needle puncture in the cornea, which rapidly extended until the entire cornea became involved, and a large portion of it in front of the pupil suppurated and sloughed off. Under appropriate treatment, however, the eye recovered, leaving a tolerably large central corneal opacity, and the cataract in very nearly the same condition as before the operation. Inasmuch as the operation upon the other eye was eminently successful, nothing more was done to the left; although it was evident that a large iridectomy and the extraction of the lens would in all probability, even here, succeed in a restoration to more or less perfect vision.

Profiting by my experience with the right eye, I made Graefe's linear extraction of the cataract upon the left, with perfect success in every particular, nothing untoward occurring to hinder the rapid and normal healing of the wounds.

The successful termination of this operation did away with any necessity for further interference with the other eye, although, as before remarked, the success of the second operation on the right eye was almost assured.

When the patient was dismissed, about two weeks afterwards, with a double convex No. 4 (cataract) glass, he could count fingers at a distance of thirty feet without hesitation, and could see quite small objects close to him distinctly with cataract glass No. 2½. Never having learned his letters, the usual test with test types could not be gone through with. The ability to count fingers at thirty feet would make his vission about equal to one-third (or probably a little more) of the normal vision.

The gratitude of this young man, from whom all nature had been hidden for twenty-two years, and whose eyes were now open to a new world, was truly gratifying; and it was amusing as well as instructive to observe him examine and study an object which he saw distinctly, but could only recognize it after laying his fingers upon it, so long had he been accustomed to carrying his eyes in his hands, as it were. Practice makes perfect in most things, and so will it in time enable him to transfer this power of recognition back to its original seat.

Needle operations through the cornea sometimes result unfavorably by suppurative inflammation of the cornea taking place as a result of the puncture. Where the needle is entered through the sclerotic and the lens broken up, either upon its anterior or posterior surface, destructive inflammation rarely or never ensues, and for this reason is the sclerotic puncture by far preferable and much easier of performance.

(NOTE.—Patient has been heard from since he went home, and he is now going everywhere by himself.)

CASE XLIV.—*Syphilitic Iritis, with Synechiæ and Gummy Tumors or Condylomata (Iritis Gummosa) upon the Iris.—Complete Cure.*—J. H., æt. thirty-five, Georgia, had a chancre about three years ago, which under proper treatment healed, and he had no further evidences of the disease until two months ago, when an eruption occurred upon the body, and other signs of secondary syphilis began to manifest themselves. About two weeks ago the right eye became inflamed, causing a very marked diminution of vision and giving intense pain. When he presented himself for treatment there was intolerance of light, and excessive lacrymation; the conjunctiva perfectly red and the aqueous humour very cloudy. The pupil was very narrowly and irregularly contracted, being bound down at different points to the anterior surface of the lens (posterior synechiæ), and towards the upper portion of the periphery of the iris there were two little protuberances, or tumors, about the size of a small white pea. They were covered with pigment from the iris, and appeared soft or gelatinous, as is usually the case with syphilitic or gummy tumors upon the sternum or other parts of the body. He had also all the other symptoms of iritis.

The same disease was beginning to show itself in the left eye, and would in the course of a day or two have been fully developed.

This form of iritis (iritis gummosa) is, so far as my experience goes, invariably the result of secondary syphilis, and one need not be mistaken about its character, even though there was not another symptom of the constitutional trouble. Nor need there ever be any hesitancy about the proper mode of treating such a case, for the anti-syphilitic is, par excellance, *the* treatment.

The patient was put upon iodide potassium, in 20 grain doses, combined with 1-20 gr. bi-chlor. mercury, three times daily.

Mercurial inunctions were also used, one drachm of the ointment being rubbed every night into different parts of the body, so as to avoid rubbing the same parts too often, which might and sometimes does, set up a sort of dermatitis.

Locally a three-grain solution of atropine was dropped into the eyes every three or four hours, which succeeded in breaking up some of the adhesions between the iris and lens in the right eye, and widely dilated the pupil in the left. Ice was applied at intervals over the lid, and blood was taken from the temples by means of the artificial leech (Heurteloup's.) Later a fly-blister was placed behind each ear. He was kept in a dark room while the photophobia lasted.

Under the above treatment the inflammation rapidly subsided, and the tumors upon the iris disappeared. There remained some of the posterior synechiæ even after the entire subsidence of the disease, but the vision was very little affected by them.

The skin eruption did not altogether disappear until after the expiration of about four weeks, up to which time the treatment was continued.

(Note.—Have recently seen the patient, and he is now not at all inconvenienced by the adhesions, which are the only evidences of the formerly existing disease.)

Case XLV.—*Foreign Bodies in the Ear (External Auditory Canal.)—Removal by Syringing.—Remarks.*—Lucy A., æt. forty-eight years, presents herself, with the history that she let slip a medium-sized pea into the right ear a few days ago, which is now giving her some pain, and has induced considerable deafness. The case, though quite simple, is brought before you to demonstrate how *all* foreign bodies in the ear should be removed if possible. An examination with the mirror and ear speculum discovered the pea firmly wedged in the auditory canal, just in front of the membrana tympani; and a little more pressure on the part of her various friends, who had been making many efforts, with as many instruments, to extricate it, would have thrown it immediately in contact with the drum head, and probably have produced serious inflammation of the parts. These ineffectual efforts to remove it have caused the canal to become swollen, the walls having been scratched at various points. By the use of a large-sized syringe, which held sufficient water to afford a constant stream for a few moments, the ear was syringed, and after a few trials the pea was brought out. The patient was relieved at once, and had no further trouble.

Be sure, when consulted by a person supposed to have a foreign body in the ear, to make a careful and thorough examination of the ear with mirror and speculum, and thus satisfy yourself first and foremost whether really there is anything in the canal or not; for patients will often insist that there is a bug, a pea, a button, or something of the kind in the ear, when upon examination you will find the canal entirely free from any and all such substances. By so doing you will spare yourselves much unnecessary labor and care and your patient much needless and perhaps barbarous pain.

But a short time since I was called late at night to see, in consultation, a lady, into whose ear a bug had flown, as the messenger stated, and the doctors in attendance had been unable to get it out. I found the patient in great agony, and her ear and the side of her face besmeared with blood. She stated that she knew the bug was in there, for she felt it fly against the ear and go in. The doctors, also, felt sure of its presence in the canal. "For," said one of them, "every time I introduce this instrument, (a slender piece of iron, with a minute sharp-edged spoon on the end of it) and move it a little over the bottom of the ear, I have a feeling in my hand and hear a sound as if scratching on the wing or back of a bug, but we can't get it out." With milk-warm water I cleansed the ear, watching carefully for some foreign substance in the water as it flowed out, but saw nothing more than clots of blood. Then with my mirror and speculum I examined the ear and found, not a bug, but the membrana tympani torn and lacerated in every imaginable direction, and also the walls of the canal deeply incised.

If the bug had gone into the ear at all, it had in all probability escaped before the doctors reached her, but they took it for granted that she had a bug in her ear, because she said so, and went to work accordingly with the spoon; each effort with which tore through the tense drum head, and imparted to the hand and ear the sensation and sound as if the instrument was passing over the back of a bug. The woman suffered much pain, and now has very defective hearing.

In nearly every instance you will succeed in removing a foreign body from the ear by the free use of the syringe, which by all means should be earnestly and persistently used before resorting to any other process. Pull the auricle upwards and a little backwards, so as to straighten the canal, and then throw the water slightly forward and downward, in which direction the canal runs. No matter how tightly the body may be impacted, if the water can pass behind it, which is almost invariably the case, as it returns everything is washed out before it. Only after thorough and repeated trials with the syringe have failed, is it justifiable to use any other instrument whatever; for in so doing incalculable and irreparable damage may be done by wounding the drum head or breaking and dislocating some one or all of the ossicles, etc. No damage can result from the use of the syringe if properly used.

Case XLVI.—*Syphilitic Tumors within the Eye-ball, producing loss of sight, with intense pain, and threatening the sound eye. Enucleation of ball. Recovery.*—Samuel S., æt. twenty-seven years; Georgia; has been suffering for some time from the effects of secondary syphilis in its worst and most typical form. For the past four weeks the pain has been gradually increasing, and the vision gradually diminishing in the right eye; opiates no longer afford any relief from the constant pain in the ball. When first examined with the ophthalmoscope, two small growths, each about the size of a large buck-shot, were seen growing from the temporal side of the choroid, and just behind the ciliary bodies. Upon each subsequent examination, with an interval of a week or ten days, the tumors had enlarged considerably, and finally made their appearance externally upon the sclerotic, immediately over the ciliary bodies and just behind the outer corneo-sclerotic junction, when meeting but little resistance, they grew very rapidly indeed. The left eye was beginning to show marked symptoms of being sympathetically diseased, and to check this, as well as put a stop to the constant and severe pain in the right, the enucleation of the latter was advised, since the vision was irretrievably lost.

Chloroform was administered, and the operation for enucleation was made in the usual manner, special care being taken to leave the parts surrounding the ball in as nearly their normal position as possible. After the hemorrhage had ceased a threaded needle was passed alternately in and out through the conjunctiva, around the entire edge of the circular opening from which the diseased ball came, so that when the two ends of the thread were pulled upon, the conjunctival opening closed up, as the mouth of a tobacco-pouch when the strings are drawn. By this means the parts are more accurately adjusted, and heal more rapidly, and afford the very best stump for wearing an artificial eye, which can be moved from side to side just as the natural eye, but not to the same extent.

The eye was first dressed after forty-eight hours (my usual practice in such cases), after which the patient was directed to wash the parts several times daily himself, and keep them clear of matter. The string closing the conjunctival opening, was removed after the fourth day, and the patient dismissed at the expiration of a week. The sympathetic symptoms disappeared from the other eye, and the constitutional disease having been treated in the meantime, he is now, to all appearances, sound and well.

Case XLVII.—*Abscess of the Cornea. Recovery.*—J. F. S. Grantville, Georgia; æt. twenty-five years; presents himself for treatment of corneal abscess of several weeks standing. When first seen, in the centre of the right cornea there existed a small opaque spot, presenting a deep grayish appearance; the eye was very red, with great photophobia and lacrymation, and there was constant suffering in and around the ball. The grayish spot gradually increased to the size of an ordinary small white button, became perfectly circumscribed, and assumed a yellowish hue, just as a drop of pus would look under a very thin, clear glass. It could be distinctly seen that the pus was underneath the external layers of the cornea, the anterior lamellæ over the abscess being slightly in advance of the surrounding portions.

He had received no blow upon the eye, nor was there any other apparent cause for the abscess, since the patient was a stout, robust man, in perfect health. He was, however, put upon some constitutional treatment, viz: iodide potassium and bi-chlor. mercury, and a tonic of iron, quinine, strychnine, and arsenic, with the view of putting his system in as good a condition as possible to withstand the effects of the disease. Locally, a solution of atropine (grs. ij. to ℥j.) was dropped into the eye every three or four hours, and the pupil kept widely dilated. Just as often, a small piece of a salve composed of the following ingredients, was rubbed over the eyebrow and corresponding temple:

℞.—Ext. belladonnæ, . . . . . gr. xvi.

Hyd. præcip. albi., . . . . gr. xij.

Ung. aquæ rosæ, . . . . ʒij.

M. and ft. ung.

The eye was bandaged, and at intervals of four or five hours cloths dipped in hot water were laid upon the bare lids, frequently changing them so as to keep the applications hot. As nearly as practicable, he was kept in a dark room.

The pus was steadily absorbed, the external layers of the cornea not giving way at all, and at the end of five or six weeks he was pronounced cured. Several months later, from exposure to severe weather, the disease returned, though not so violently, but gave way under the same treatment. The cornea is now quite clear, and he is not at all inconvenienced by the long existence of the inflammation.

If the abscess had shown a tendency to spread, and thus affect more of the corneal tissue, the best treatment would have been to lay it open by cutting through the entire depth of the cornea, bringing the knife out upon each side of the abscess in the sound tissue. This would in all probability have stopped its progress, just as the opening of an ordinary abscess in any other part of the body checks its advance. Should the abscess break of its own accord, through the external corneal layers, it leaves an ulcer, which after long duration heals up, but leaves behind a cicatrix, which almost invariably affects the vision to greater or less degree.

